# Loss of Sympathetic Nerves in Spleens from Patients with End Stage Sepsis

**DOI:** 10.3389/fimmu.2017.01712

**Published:** 2017-12-06

**Authors:** Donald B. Hoover, Thomas Christopher Brown, Madeleine K. Miller, John B. Schweitzer, David L. Williams

**Affiliations:** ^1^Department of Biomedical Sciences, Quillen College of Medicine, East Tennessee State University, Johnson City, TN, United States; ^2^Center of Excellence in Inflammation, Infectious Disease and Immunity, Quillen College of Medicine, East Tennessee State University, Johnson City, TN, United States; ^3^Department of Pathology, Quillen College of Medicine, East Tennessee State University, Johnson City, TN, United States; ^4^Department of Surgery, Quillen College of Medicine, East Tennessee State University, Johnson City, TN, United States

**Keywords:** noradrenergic, cholinergic, human, spleen, sepsis, innervation, remodeling

## Abstract

The spleen is an important site for central regulation of immune function by noradrenergic sympathetic nerves, but little is known about this major region of neuroimmune communication in humans. Experimental studies using animal models have established that sympathetic innervation of the spleen is essential for cholinergic anti-inflammatory responses evoked by vagal nerve stimulation, and clinical studies are evaluating this approach for treating inflammatory diseases. Most data on sympathetic nerves in spleen derive from rodent studies, and this work has established that remodeling of sympathetic innervation can occur during inflammation. However, little is known about the effects of sepsis on spleen innervation. Our primary goals were to (i) localize noradrenergic nerves in human spleen by immunohistochemistry for tyrosine hydroxylase (TH), a specific noradrenergic marker, (ii) determine if nerves occur in close apposition to leukocytes, and (iii) determine if splenic sympathetic innervation is altered in patients who died from end stage sepsis. Staining for vesicular acetylcholine transporter (VAChT) was done to screen for cholinergic nerves. Archived paraffin tissue blocks were used. Control samples were obtained from trauma patients or patients who died after hemorrhagic stroke. TH + nerves were associated with arteries and arterioles in all control spleens, occurring in bundles or as nerve fibers. Individual TH + nerve fibers entered the perivascular region where some appeared in close apposition to leukocytes. In marked contrast, spleens from half of the septic patients lacked TH + nerves fibers and the average abundance of TH + nerves for the septic group was only 16% of that for the control group (control: 0.272 ± 0.060% area, *n* = 6; sepsis: 0.043 ± 0.026% area, *n* = 8; *P* < 0.005). All spleens lacked cholinergic innervation. Our results provide definitive evidence for the distribution of noradrenergic nerves in normal human spleen and the first evidence for direct sympathetic innervation of leukocytes in human spleen. We also provide the first evidence for marked loss of noradrenergic nerves in patients who died from sepsis. Such nerve loss could impair neuroimmunomodulation and may not be limited to the spleen.

## Introduction

The central nervous system (CNS) and immune system play essential roles in maintaining homeostasis, and there is extensive evidence for crosstalk between these systems (1–5). Mediators generated during injury or inflammation stimulate sensory pathways to the brain, which responds by activating the hypothalamic–pituitary–adrenal axis and efferent autonomic neural pathways. Activation of the sympathetic nervous system (SNS) evokes well-known cardiovascular and metabolic responses aimed at addressing threats to homeostasis. As the sole source of efferent neural input to primary and secondary lymphoid tissues, the SNS also plays an essential role in CNS modulation of immune function (1, 6, 7). These effects on immune function can be indirect through modulation of blood flow or direct through stimulation of adrenergic receptors on leukocytes (1).

The spleen is the largest secondary lymphoid tissue and a major hub of innate and adaptive immune responses, making it a prime target for regulation by the SNS (1, 3, 8). Experimental studies have shown that the spleen is a major contributor to the exaggerated inflammatory response that occurs in sepsis, trauma, and burn injuries (3). Furthermore, adverse cellular and functional remodeling of the spleen contributes to immunosuppression in patients who die from sepsis and multiple organ failure (9). Accordingly, understanding neuromodulatory mechanisms that operate within the spleen has significant translational value. Neuroanatomical studies, done primarily with small animal models, have shown that sympathetic nerves enter the spleen with major vessels and distribute throughout the arterial vasculature (1, 10). Many sympathetic nerve fibers travel into the periarterial lymphatic sheath (PALS) where some occur in close apposition to T cells, B cells, and macrophages, providing a structural basis for direct neuromodulation of immune function (10–13). Diffusion of catecholamines from their release sites would also affect more leukocytes *via* so-called volume transmission (1, 2, 5, 12).

Both the spleen and its sympathetic nerves have essential roles in the cholinergic anti-inflammatory pathway (3, 4, 8). Activation of splenic sympathetic nerves by reflex mechanisms or vagal nerve stimulation triggers release of norepinephrine (NE), which stimulates β_2_-adrenergic receptors on cholinergic T cells, causing release of acetylcholine (ACh). Then, ACh elicits an anti-inflammatory response mediated by α7 nicotinic ACh receptors on macrophages (3, 8). In addition to this mechanism, there is substantial evidence that catecholamines can affect the functions of macrophages, dendritic cells, T_regs_, and B cells by stimulation of surface adrenergic receptors (1).

The translational potential for neural modulation of immune function in disease is already being tested in ongoing clinical trials of vagal nerve stimulation, which activates sympathetic input to the spleen (3). However, most studies that defined the localization of sympathetic nerves in spleen used animal models, and our knowledge regarding sympathetic innervation of adult human spleen is very sparse (14). Furthermore, experimental studies provide evidence that sympathetic innervation of the spleen can change with age and inflammatory disease (5, 15–17), but analogous human data are lacking. To address these gaps in knowledge, we used immunohistochemistry to evaluate the presence and localization of noradrenergic sympathetic nerves in sections of control spleens and spleens from patients with end stage sepsis. Additional immunostaining was done for the vesicular acetylcholine transporter (VAChT) to evaluate the presence of cholinergic nerves in the spleen.

## Materials and Methods

### Human Tissue Samples

All tissue sections evaluated in this study were obtained from paraffin-embedded samples graciously provided by Dr. Richard Hotchkiss at Washington University. Many of these samples were used in prior studies of sepsis by Hotchkiss and his colleagues (9). All of these studies were approved by the Washington University or St. John’s Hospital Human Research Protection Office, with informed consent, which included permission to collect tissue for research purposes, provided by a next-of-kin in accordance with the Declaration of Helsinki. Spleen samples from septic patients who died in the intensive care unit were obtained by immediate, limited autopsy performed at bedside after obtaining permission from next-of-kin. Control samples were obtained during surgical removal of the spleen to control bleeding after upper body trauma, from organ donors with permission from next-of-kin to collect tissue for research purposes, and postmortem from patients with subarachnoid hemorrhage. Clinical information provided to us and comments on hematoxylin and eosin (H&E) stained sections of spleens are summarized in Table 1.

**Table 1:** Patient profiles.

Group	Gender	Age	Clinical situation	Hematoxylin and eosin histology
Controls	Female	74	Subarachnoid hemorrhage	Some white pulp (WP) loss, no germinal centers (GC)
	Female	51	CHF, COPD, subarachnoid hemorrhage	Some WP loss, rare GC
	Male	NA	Trauma	Normal
	Male	NA	Trauma	Normal
	Male	NA	Trauma	Normal
	Male	NA	Trauma	Normal, no GC

Septic	Male	63	Pancreatitis, multiple intra-abdominal abscesses, VAP, renal failure	Marked WP loss, no GC, much hemosiderin
	Female	87	S/P pelvic exenteration for cervical cancer, bacteremia w/respiratory distress, hypotension	Poorly defined WP, no GC
	Female	70	S/P femoral popliteal bypass, anemia, massive MI, vasopressors, ischemic bowel on postmortem	Decreased WP, rare GC
	Female	87	Ischemic colitis, septic about 2 days	Decreased WP, rare GC
	Male	80	Upper GI bleed and peritonitis	Marked WP loss, no GC
	Male	62	Septic w/dead gut	Marked WP loss, no GC
	Male	60	Septic w/peritonitis and obese	Some WP, empty sinusoids
	Female	NA	Influenza complicated by bacterial pneumonia	Decreased WP

### Tissue Processing and Immunohistochemistry

5 µm sections of paraffin-embedded spleens and a control sample of intestine were cut using a Leica RM2135 microtome, collected on charged slides, and stained with H&E or processed for bright field immunohistochemistry using standard procedures (18, 19). Briefly, sections were deparaffinized, rehydrated, and incubated in citrate buffer (pH 6.5) for 20 min at 92°C for antigen retrieval. Sections were washed with 0.1 M phosphate-buffered saline (PBS, pH 7.3), permeabilized in PBS containing 0.4% Triton X-100 and 0.5% bovine serum albumin, and treated with 1% H_2_O_2_ in PBS for 15 min. After additional washing and blockade in PBS containing 5% normal goat serum, 1% BSA, and 0.4% Triton X-100, the sections were incubated with primary antibody overnight. Primary antibodies were rabbit anti-tyrosine hydroxylase (TH) (1:1,000, Pel-Freez Cat. # P40101; Rogers, AR, USA) or rabbit anti-VAChT (1:1,000, Cat. # 139103; Synaptic Systems, Göttingen, Germany). Further processing was done using a Rabbit ABC kit (Vector Laboratories), and Vector ImmPact VIP chromogen (Vector Laboratories) was used to produce a purple reaction product at sites of antigen localization. For some sections from control spleens, Vector ImmPact DAB was used as the chromogen to stain TH nerves brown, and hematoxylin was used as a counterstain to visualize nuclei. Slide-mounted sections were viewed using an Olympus BX4 microscope, and digital images were obtained using an attached Olympus Q-Color 3 digital camera and Q-Cap Pro 7 software.

### Analysis of TH Nerve Fiber Abundance

We performed semi-quantitative and quantitative analyses to evaluate the abundance of TH-stained nerves fibers in spleen sections. Both analyses were performed in a blinded fashion. Semi-quantitative evaluation of TH staining was performed by a neuropathologist using the following grading scale: 0—no staining, 1—staining just around large vessels near the capsule, 2—staining around large vessels and trace staining in parenchyma including small vessels, and 3—staining around large vessels and in parenchyma.

Quantitative evaluation of TH nerve fiber abundance was performed using ImageJ software (U.S. National Institutes of Health, Bethesda, MD, USA). Slides were viewed with a 20× objective and scanned in a standard pattern, collecting non-overlapping images of fields containing at least one blood vessel. This process resulted in 15–45 images per section, depending on the area of the section. These images were analyzed in a blinded fashion by another individual to determine the % of each field that contained TH + nerve fibers. Resulting data are presented as % area.

### Statistical Analyses

Statistical analysis of nerve abundance data was performed using GraphPad Prism version 6.00 (GraphPad Software, La Jolla, CA, USA). Semi-quantitative data for control and sepsis groups were compared by non-parametric analysis using a two-tailed Mann–Whitney test for unpaired observations. Quantitative data were compared by using a two-tailed *t*-test for unpaired samples. A *P* value less than 0.05 was considered significant. Data are presented as mean ± SD.

## Results

Clinical and general histological details of patients from whom spleens were obtained retrospectively for examination are presented in Table 1. There were six control specimens: these were obtained from four trauma patients and two who died after subarachnoid hemorrhage whose spleens were obtained at autopsy. The spleens of eight patients who died with sepsis were obtained at postmortem examination. The spleens of the traumatized controls showed well-defined white pulp (WP) with numerous germinal centers (GC) and unremarkable vessels. The spleens of the controls with terminal subarachnoid hemorrhage showed well defined but less extensive WP with fewer GC. They also showed hyalinizing vascular changes that are common age-related changes at postmortem examination. The WP was greatly decreased in seven out of eight septic spleens (in the fourth it was very poorly delineated), and GC were very rare to completely lacking in all specimens. One septic spleen showed abundant hemosiderin deposition.

**Figure 1:**
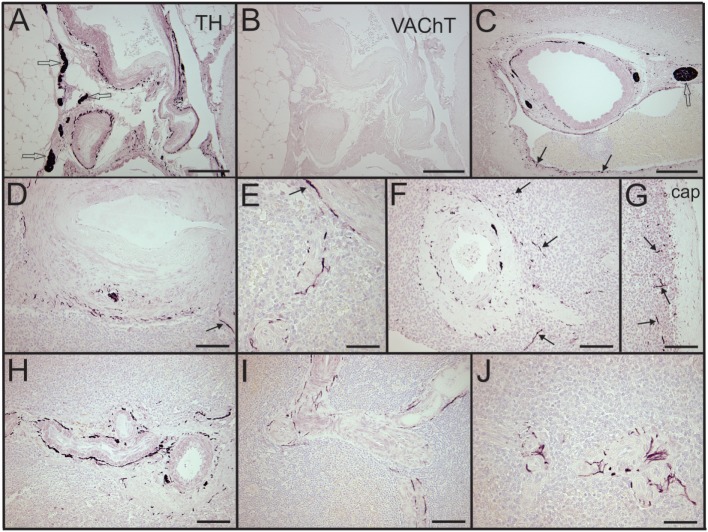
Control human spleens had abundant tyrosine hydroxylase (TH) + sympathetic nerves associated with the vasculature and lacked cholinergic innervation. **(A,B)** Images showing noradrenergic (TH+) nerve fibers and bundles around splenic artery and branches **(A)** and absence of cholinergic (VAChT) nerves in an adjacent section **(B)** from a 74-year-old female. **(C)** Low magnification image of TH + nerve fibers and bundles around a trabecular artery in a spleen section from a male trauma patient. Arrows indicate TH + nerve fibers around an adjacent large vein. **(D,E)** TH + fibers associated with central artery and smaller branches in the same 74-year-old female. Nerve fibers are localized to adventitia and adventitia-media border. Arrow in **(D)** indicates nerve around branch of central artery shown at higher magnification in **(E)**. **(F)** Sympathetic nerves around a central artery and branch from another trauma patient. Arrows indicate some TH + nerve fibers that entered the periarterial lymphatic sheath. **(G)** Same patient had TH + nerve fibers (some indicated by arrows) in lymphatic tissue under the capsule. **(H,I)** TH + fibers around small, branching arteries in sections from first trauma patient **(H)** and from a 57-year-old female **(I)**. **(J)** Periarteriolar TH + nerve fibers in spleen section from a 74-year-old female. TH + nerve fibers extend a short distance into surrounding lymphatic tissue. Open arrows indicate some TH + nerve bundles. Scale bars: 250 µm **(A–C)**; 100 µm **(D,F–I)**; 50 µm **(E,J)**.

**Figure 2:**
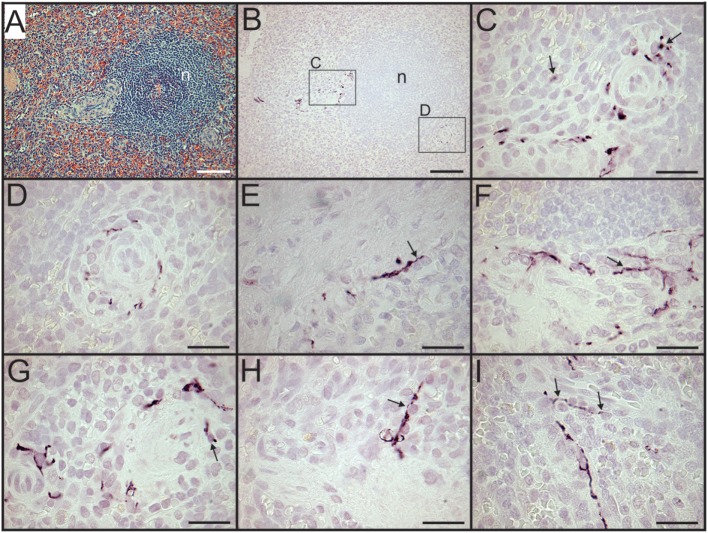
Sympathetic nerves in perivascular lymphatic tissue of control spleens have close association with leukocytes. **(A,B)** Hematoxylin and eosin stain of small arteries associated with a splenic nodule (*n*) in section from a trauma patient **(A)** and tyrosine hydroxylase (TH) + nerve fibers around same vessels in adjacent section **(B)**. Small boxes indicate regions shown at higher magnification **(C,D)** to demonstrate close apposition of sympathetic nerves with leukocytes. **(E,F)** Close apposition of sympathetic nerves with perivascular leukocytes (arrows) in spleen sections from female patients who were 74 and 57 years old, respectively. **(G–I)** Close apposition of TH + nerve processes with perivascular leukocytes (some indicated by arrows) in spleen section from another trauma patient. Scale bars: 100 µm **(A,B)**; 25 µm **(C–I)**.

**Figure 3:**
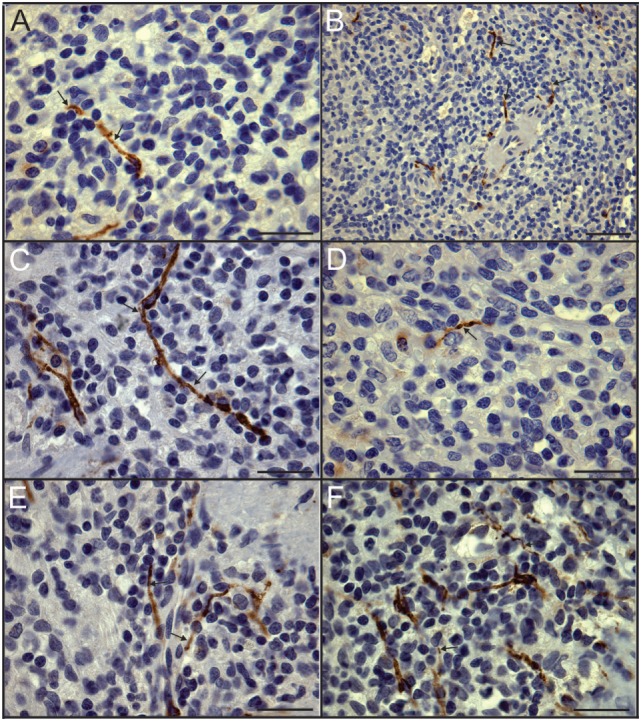
Close association of tyrosine hydroxylase (TH) + nerves and leukocytes demonstrated in dual stained sections. **(A–F)** High magnification images of control spleen sections with TH + nerves stained brown using DAB and nuclei stained blue with hematoxylin to enhance identification of leukocytes. Arrows indicate some TH + nerves in close apposition to leukocytes. Scale bars: 25 µm **(A,C–F)**; 50 µm **(B)**.

Sympathetic nerves were identified by immunostaining spleen sections for TH, the rate-limiting enzyme for synthesis of the noradrenergic neurotransmitter, NE. Such nerves were prominent in all control patients, with no qualitative difference between TH staining of spleens obtained at surgery compared to those obtained at autopsy. TH + nerves entered the spleen in large bundles associated with penetrating vessels (Figure 1A) and continued to be present in bundles of decreasing size along the arterial vasculature within the parenchyma of the spleen (Figures 1C,D,F,H). The splenic artery, trabecular arteries, central arteries, and smaller branches down to arterioles were surrounded by individual nerves fibers present at the adventitia-media border and in the adventitia (Figures 1A,C–F,H–J). Subscapular TH + nerve fibers were abundant in sections from one trauma patient, who had the most robust sympathetic innervation (Figure 1G). However, none of the control patients had sympathetic nerves within the capsule (Figure 1G). While the vast majority of sympathetic nerves in the control spleens were associated with the arterial vasculature, several TH + nerve fibers extended short distances into the PALS (Figures 1F, 2 and 3). Sympathetic nerves were not present in follicles or GC (Figures 2A,B). Examination of perivascular regions of control human spleens at high resolution with a 100× oil objective revealed extremely close apposition of nerve fibers and varicosities with leukocytes (Figures 2C-I and 3).

**Figure 4:**
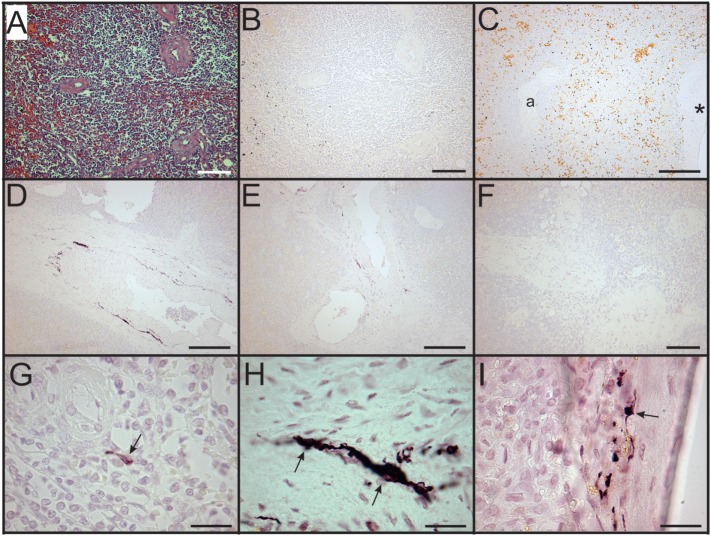
Loss of sympathetic nerves in spleens from patients who died with sepsis. **(A,B)** Hematoxylin and eosin stain **(A)** and tyrosine hydroxylase (TH) stain **(B)** of spleen section from 63-year-old male. Note absence of TH + nerve fibers around arteries. **(C)** Absence of TH + nerves around large artery (*) and smaller artery (a) in spleen section from an 87-year-old female patient. Sections from both of these patients lacked sympathetic nerves. **(D–F)** Images showing reduced abundance of periarterial TH + nerve fibers in spleen from a 70-year-old female patient who died with sepsis. Large **(D)** and intermediate **(E)** diameter arteries show marked reduction of TH + nerve fibers compared to control patients and lacked large TH + nerve bundles. Smaller arteries **(F)** lacked TH + nerve fibers in this patient. **(G)** Solitary axon retraction ball in white pulp. **(H)** Tortuous reactive axonal profile with highly variable axonal diameter. **(I)** Axonal swelling where apparently intact axon profiles enter and leave zone of interrupted axonal transport. Scale bars: 100 µm **(A,B,F)**; 250 µm **(C–E)**; 25 µm **(G–I)**.

**Figure 5:**
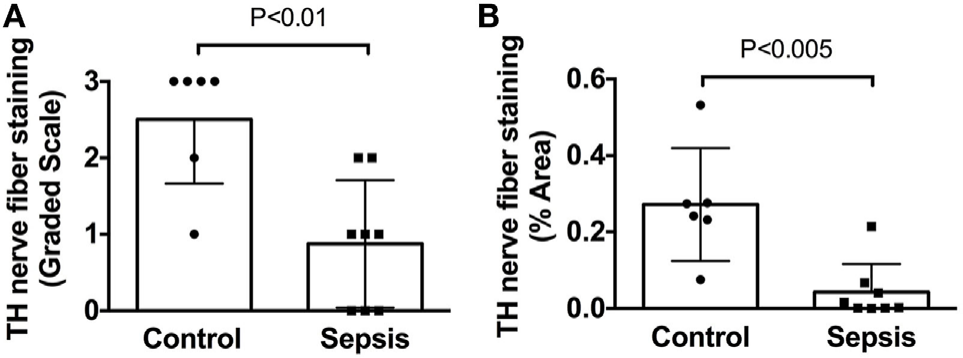
Quantification of tyrosine hydroxylase (TH) nerve fiber staining shows a significant deficit in spleens from patients with end stage sepsis. **(A)** Semi-quantitative evaluation of TH nerve fiber abundance was performed using a grading scale ranging from 0 to 3 as described in Section “Materials and Methods.” **(B)** Quantitative evaluation of TH nerve fiber abundance was performed, as described in Section “Materials and Methods,” using ImageJ to measure the % area occupied by TH + nerve fibers. Both methods showed a significant reduction of TH + nerve fibers in spleen samples from septic patients compared to control patients. Data in panel **(A)** were evaluated using a two-tailed Mann–Whitney test for unpaired samples (*n* = 6 for control and *n* = 8 for septic; *P* = 0.0077). Data in panel **(B)** were evaluated using a two-tailed *t*-test for unpaired samples (*P* = 0.0024). Values are mean ± SD.

In sharp contradistinction to control spleens, TH + nerve bundles and fibers were lacking in four of the eight septic spleens and greatly reduced in number in the other four (Figure 4). Residual TH + nerves in the latter spleens were localized primarily to larger vessels, where they occurred in much smaller bundles. Noradrenergic nerve fibers were uncommon in smaller vessels and seldom extended into perivascular regions of the septic spleens. Abnormal axonal profiles were identified among the residual TH + nerve fibers in spleens from septic patients (Figures 4G–I). Sympathetic nerve abundance in spleens from control and septic patients was evaluated and compared semi-quantitatively using an arbitrary grading scale and quantitatively by measuring area occupied by TH + neural elements. Both methods demonstrated significant decreases in TH + nerve fiber abundance in spleens from septic patients compared to controls (Figure 5). Quantitative evaluation of TH + nerve staining showed that the average abundance of TH + nerves in the septic group was only 16% of that for the control group (control: 0.272 ± 0.060% area, *n* = 6; sepsis: 0.043 ± 0.026% area, *n* = 8; *P* < 0.005).

**Figure 6:**
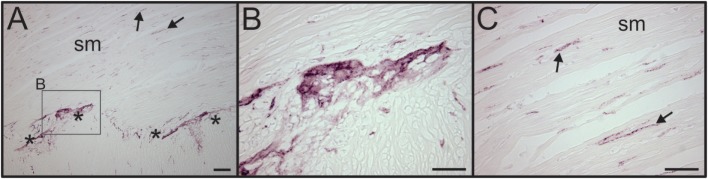
VAChT antibody labels cholinergic nerve fibers in human intestine. **(A)** Low magnification image showing the presence of VAChT + cholinergic nerve fibers in a section of human intestine that was stained along with spleen sections. Asterisks indicate a ganglionated nerve plexus. Boxed region contains a ganglion shown at high magnification in panel **(B)**. Arrows indicates two of many VAChT nerve fibers that innervated intestinal smooth muscle (sm). **(B)** VAChT + nerve fibers surrounding an enteric ganglion. **(C)** VAChT + nerve fibers in sm region shown at higher magnification. Scale bars: 100 µm **(A)**; 50 µm **(B,C)**.

We also evaluated adjacent sections of spleen for the presence of VAChT, which is a specific marker for cholinergic nerves. No VAChT + nerves fibers or bundles were detected in any of the spleen sections (Figure 1B), but prominent cholinergic nerve fibers were detected in a section of human intestine processed in the same experiment (Figure 6).

## Discussion

Sympathetic innervation of lymphoid tissues, such as the spleen, has been recognized for a long time (14, 20, 21), but the precise localization of noradrenergic nerve fibers within these tissues has only been revealed with modern neuroanatomical techniques over the past three decades. The vast majority of this work was performed using animal models, and data obtained from human lymphoid tissue are sparse (14). Our findings address this gap in knowledge by providing a detailed localization of noradrenergic nerve fibers in normal human spleen, and we also present evidence for sympathetic remodeling in patients with end stage sepsis. These findings are especially significant given recent advances in our understanding of functional interactions between sympathetic nerves and immune cells in the spleen.

We observed that noradrenergic nerves are abundant throughout the arterial vasculature of control human spleens but absent in the splenic capsule. These neuroanatomic findings are consistent with functional data obtained from early experimental studies of isolated perfused human spleens (20, 22). The latter work showed that stimulation of postganglionic sympathetic nerves to the human spleen or close arterial injection of NE or epinephrine had only minor effects on spleen volume but caused graded vasoconstriction. This contrasts with dogs, which showed vasoconstriction and a large reduction of spleen volume due to contraction of smooth muscle in the capsule (20, 23).

Our immunohistochemical findings provide the first, high-resolution evidence for localization of sympathetic nerves in the splenic vasculature of adult humans lacking major systemic pathology. This builds on previous work, which used a fluorescence histochemical method, to visualize catecholamine-containing nerves in spleens obtained from patients with gastrointestinal pathology, primarily advanced gastric cancer (14). The latter study also reported the presence of cholinergic nerves in the human spleen based on staining for acetylcholinesterase, which is now known to be a non-specific marker that also labels some non-cholinergic nerves. Our staining for the VAChT, a selective cholinergic marker and protein required for cholinergic neurotransmission, did not detect cholinergic nerves in the spleen but did show parasympathetic nerves in a control sample from human intestines, which was processed with the spleen sections. Thus, our findings for human spleen support the conclusion, based on work with other species, that the spleen lacks cholinergic parasympathetic innervation (1, 3, 6).

While a vast majority of sympathetic nerves in the human spleen were associated with the vasculature, we observed several smooth and varicose TH + nerve fibers that entered periarterial regions of control spleens where they occurred in close proximity to leukocytes. To the best of our knowledge, this is the first demonstration of TH + nerves within leukocyte regions of the human spleen. We did not examine the phenotype of leukocytes that occurred in close apposition to TH + nerves in the human spleen, but other investigators identified T cells, B cells, and macrophages associated with noradrenergic nerve fibers in rodent spleens (10–13). It is important to note that most of these nerve fibers traveled relatively short distance into the parenchyma of the spleen. This pattern differs from results for rats, mice, and rabbits, which have dense noradrenergic innervation throughout the white pulp (24). Ultrastructural studies of rat spleen have demonstrated that some leukocyte and axonal membranes are separated by as little at 6 nm at points of close apposition (10). However, the abundance of such “synapse-like” structures was not reported, and this mode of transmission would be atypical for the autonomic nervous system where varicosities are separated from effector tissues by gaps ranging from about 20 nm to about 2 µm (25). Direct communication between noradrenergic nerve fibers and cholinergic T cells in the spleen was suggested to play a mechanistic role in the vagal anti-inflammatory pathway (13). However, recent neuroanatomical studies found that less than 5% of cholinergic T cells occurred within 8 µm of a TH + axon (12). These investigators proposed that communication between noradrenergic nerves and cholinergic T cells in the spleen is more likely to occur by “volume transmission,” where the sphere of nerve influence is determined by diffusion of NE. Since most of the noradrenergic nerves that we identified in human spleen were localized in and close to blood vessels, diffusion of NE into surrounds PALS could provide a major mechanism for neuroimmune regulation in humans.

Much of the knowledge that we have regarding effects of adrenergic neurotransmitters on the immune system comes from *in vitro* or *ex vivo* studies of immune cells and studies of immune cell lines (1, 5). This work has shown that virtually all types of immune cell express adrenergic receptors, with the specific receptor types showing plasticity related to factors such as time, activation state, and duration of exposure to agonist. Adrenergic agonists can affect both innate and adaptive immune responses with activation of β_2_-adrnergic receptors on immune cells often mediating anti-inflammatory effects and α_2_ receptors mediating pro-inflammatory effects. Knowledge about specific neuronal–immune cell interactions in the spleen was sparse until the recent discovery of the cholinergic anti-inflammatory pathway. This work has established that noradrenergic nerves and cholinergic T cells in the spleen are crucial for the anti-inflammatory response to vagal nerve stimulation in endotoxemia and several other inflammatory states (3, 4, 8).

Sympathetic effects on spleen function would undoubtedly suffer during sepsis based on the deficiency of TH + nerves we detected in pathological samples. We do not know why sympathetic nerves are lost in the spleen of patients with end stage sepsis or if this change is restricted to the spleen. However, there is abundant evidence for plasticity of noradrenergic sympathetic nerves in human inflammatory diseases and animal models (5, 16, 26–28). This work suggests that nerve loss could be due to changes in the local environment that (1) decrease availability of neurotrophic factors that support sympathetic neurites, (2) increase the production of inflammatory cytokines and/or nerve repellant chemicals, and/or (3) generate toxic free radicals.

**Figure 7:**
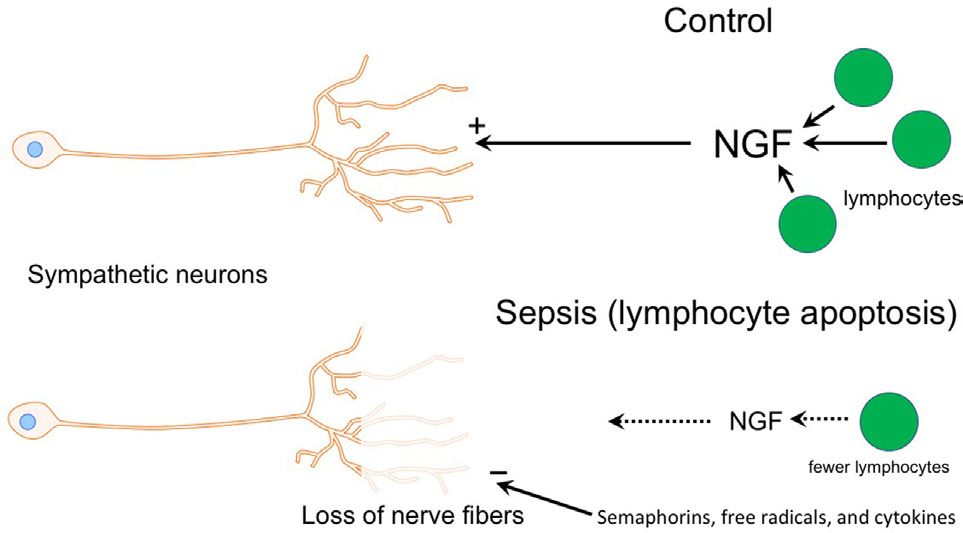
Diagram illustrating potential mechanisms contributing to loss of sympathetic nerves in end stage sepsis. Top panel shows normal condition (control) where sympathetic innervation is maintained by nerve growth factor (NGF), which is most likely supplied by T and B cells. The bottom panel depicts changes in sepsis that could contribution to the loss of sympathetic nerves. Loss of lymphocytes in the septic spleen would decrease the supply of NGF, which is needed to maintain sympathetic nerves. Semaphorins, which are nerve repellants, could be generated in the septic spleen and cause retraction of sympathetic nerves through their interaction with the neuropilin-2/plexin A2 receptor complex located on tyrosine hydroxylase (TH) + nerve fibers. Toxic free radicals and inflammatory cytokines, generated locally during sepsis, might contribute to degeneration of TH + nerve fibers.

Sympathetic neurons require stimulation by nerve growth factor (NGF) for survival during development and for maintenance of sympathetic innervation throughout life (29). NGF is a target-derived neurotrophic factor that is secreted by adrenoceptive cells in organs and tissues innervated by noradrenergic nerves. Recent studies have shown that NGF is synthesized in and released by several types of immune cells including T cells, B cells, and macrophages (30–32). Since T and B cell abundance in the spleen is reduced substantially during sepsis due to apoptosis (9, 33, 34), it is reasonable to conclude that the supply of NGF to support sympathetic innervation would likewise be reduced (Figure 7). Previous studies identified significant loss of noradrenergic nerves and reduction of NE content in spleen and other lymphoid tissue of old F344 rats (16, 35), and several mechanisms have been proposed as causative factors in this age-dependent loss of sympathetic nerves (17, 36–38). Deficiency of neurotrophic factors ranks among these since aging-associated loss of noradrenergic nerves in the spleen and reduction of NE content occur in parallel with signs of immunosuppression (17). Aging alone is not universally associated with loss of sympathetic nerves since noradrenergic nerves are well maintained in the spleen of several strains of mice and rats (15, 39), and we observed prominent TH + nerves in spleen from a 74-year-old control female patient. Rather, it is likely that overall health and specifically health of the immune system are critical factors.

Damage from free radicals was proposed as another contributor to age-associated sympathetic nerve loss in the spleen and lymphoid tissue of rats (38, 40, 41), and free radicals are clearly generated during sepsis. Thus, reactive oxygen and nitrogen species generated in the spleen might trigger nerve loss by direct toxic effects on TH + nerves and by toxic effects on target cells, resulting in reduced production of neurotrophic factors (Figure 7). Several studies have shown that treatment of old rats with l-deprenyl, an agent known to increase activity of scavenger enzymes and reduce oxidative stress (42), causes partial restoration of noradrenergic nerves in the spleen and improves immune function (38, 40, 41). Part of this effect may be due to reduction of free radicals but l-deprenyl might also have neurotrophic actions (43).

Previous studies reported loss of sympathetic nerve fibers in several tissues during inflammation, an effect most thoroughly studied in synovial tissue from patients with rheumatoid arthritis (RA) and in experimental models of RA (5). Upregulation of nerve repellant factors such as semaphorin 3C and 3F in inflamed joints has been implicated in the selective loss of TH + nerves in RA (26). Semaphorin molecules are important axon guidance factors during development and function by activating heteromeric receptors comprising a neuropilin ligand-binding unit and a plexin signaling unit (26, 44, 45). Immunohistochemical studies have localized neuropilin-2 to sympathetic nerve fibers in synovial tissue from patients with RA and osteoarthritis (26), so expression of semaphorin receptors by sympathetic neurons and nerve fibers continues in adults. It is interesting that remodeling of sympathetic innervation has been reported in the spleen of rats with adjuvant-induced arthritis (28). In this model, TH + nerve fiber abundance is reduced in the PALS and undergoes sprouting in the red pulp. Given the inflammatory milieu present in the spleen during sepsis, it is feasible that increased synthesis and release of semaphorins might occur and contribute to sympathetic nerve loss (Figure 7).

Inflammatory cytokines might also contribute to sympathetic nerve loss in the spleen. Excessive production of inflammatory cytokines is a major cause of pathophysiology in patients with sepsis, and some of these cytokines [i.e., interleukin-1β and tissue necrosis factor-α (TNF-α)] have been implicated in CNS neurodegeneration (46, 47). Sympathetic neurons are known to contain receptors for several cytokines (48–52), and TNF-α in particular has actions during development to promoted apoptosis and inhibit neurite growth (49, 53). However, there is no evidence for neurotoxic effects of cytokines on adult sympathetic neurons and some can actually stimulate neurite growth (50–52). Thus, it is unlikely that cytokines contribute to the TH + nerve loss in patients with end stage sepsis.

While this study establishes that sympathetic innervation of the spleen is reduced or lost in patients with end stage sepsis, the clinical impact of this deficit remains a matter of speculation. One possibility is that sympathetic nerve loss might favor immunosuppression, but it is also possible that altered immune phenotype contributes to nerve loss. In any event, loss of sympathetic nerves would preclude any effects that they might have to restore immune function in the spleen.

This study has some limitations that merit consideration. Sample size was small but adequate for this initial inquiry. More importantly, we had limited information on the clinical history of sepsis patients. Knowing the duration of sepsis would be useful since this parameter is likely to affect the magnitude of nerve loss. In this regard, we did note that one patient had sepsis for only a few days before dying, and that patient had the highest nerve abundance within the sepsis group. Age information was lacking for some of the trauma patients, but these individuals are generally younger than sepsis patients. Accordingly, we cannot eliminate the possibility that some loss of sympathetic innervation occurred with aging. Age-related loss of sympathetic nerves in spleen has been variable in animal studies, depending on strain (15, 39). Patients with sepsis often have multiple comorbidities, and we do not know if these affect sympathetic innervation of the spleen. Therefore, it would be important for future study to compare sympathetic innervation in of spleens from non-septic, elderly patients with and without comorbidities. Despite these limitations, this study has established a solid foundation that supports the translational potential for neuromodulation of immune function by noradrenergic nerves in the spleen. Further work is needed to define the phenotypes of splenocytes that receive noradrenergic contacts, the abundance of such contacts, adrenergic receptors expressed by different leukocyte populations, and plasticity of both nerves and receptors in disease.

In summary, we present definitive evidence for the distribution of noradrenergic nerves in normal human spleen and the first evidence for the presence of sympathetic nerves near leukocytes located in the PALS of human spleen. In addition, we provide the first evidence for marked loss of noradrenergic nerves in patients who died from sepsis. We speculate that loss of splenic noradrenergic nerves may lead to impaired neuromodulation in sepsis and this may not be limited to the spleen.

## Ethics Statement

All tissue sections evaluated in this study were obtained from paraffin-embedded samples graciously provided by Dr. Richard Hotchkiss at Washington University. Many of these samples were used in prior studies of sepsis by Hotchkiss and his colleagues (9). All of these studies were approved by the Washington University or St. John’s Hospital Human Research Protection Office, with informed consent, which included permission to collect tissue for research purposes, provided by a next-of-kin in accordance with the Declaration of Helsinki. Spleen samples from septic patients who died in the intensive care unit were obtained by immediate, limited autopsy performed at bedside after obtaining permission from next-of-kin. Control samples were obtained during surgical removal of the spleen to control bleeding after upper body trauma, from organ donors with permission from next-of-kin to collect tissue for research purposes, and postmortem from patients with subarachnoid hemorrhage.

## Author Contributions

The study was planned by DH and DW. TB, MM, and DH performed experiments. DH and JS evaluated stained sections, and DH collected images and prepared figures. DH and JS wrote the manuscript and all authors reviewed and commented on it.

## Conflict of Interest Statement

The authors declare that the research was conducted in the absence of any commercial or financial relationships that could be construed as a potential conflict of interest. The reviewer CM and handling editor declared their shared affiliation.
